# EasyPCC: Benchmark Datasets and Tools for High-Throughput Measurement of the Plant Canopy Coverage Ratio under Field Conditions

**DOI:** 10.3390/s17040798

**Published:** 2017-04-07

**Authors:** Wei Guo, Bangyou Zheng, Tao Duan, Tokihiro Fukatsu, Scott Chapman, Seishi Ninomiya

**Affiliations:** 1 International Field Phenomics Laboratory, Institute for Sustainable Agro-ecosystem Services, Graduate School of Agricultural and Life Sciences, The University of Tokyo, 1-1-1, Midori-cho, Nishitokyo, Tokyo 188-0002, Japan; guowei@isas.a.u-tokyo.ac.jp; 2 CSIRO Agriculture & Food, Queensland Biosciences Precinct, 306 Carmody Rd., St. Lucia, QLD 4067, Australia; bangyou.zheng@csiro.au (B.Z.); duantaohao@126.com (T.D.); Scott.Chapman@csiro.au (S.C.); 3 Institute College of Resources and Environmental Sciences, China Agricultural University, Beijing 100193, China; 4 Institute of Agricultural Machinery, National Agriculture and Food Research Organization, Kannondai 1-31-1, Tsukuba-shi, Ibaraki 305-0856, Japan; fukatsu@affrc.go.jp; 5 School of Agriculture and Food Sciences, Building 8117A NRSM, The University of Queensland, Gatton, QLD 4343, Australia

**Keywords:** phenotyping, digital images, plant canopy coverage ratio, field image data

## Abstract

Understanding interactions of genotype, environment, and management under field conditions is vital for selecting new cultivars and farming systems. Image analysis is considered a robust technique in high-throughput phenotyping with non-destructive sampling. However, analysis of digital field-derived images remains challenging because of the variety of light intensities, growth environments, and developmental stages. The plant canopy coverage (PCC) ratio is an important index of crop growth and development. Here, we present a tool, EasyPCC, for effective and accurate evaluation of the ground coverage ratio from a large number of images under variable field conditions. The core algorithm of EasyPCC is based on a pixel-based segmentation method using a decision-tree-based segmentation model (DTSM). EasyPCC was developed under the MATLAB^®^ and R languages; thus, it could be implemented in high-performance computing to handle large numbers of images following just a single model training process. This study used an experimental set of images from a paddy field to demonstrate EasyPCC, and to show the accuracy improvement possible by adjusting key points (e.g., outlier deletion and model retraining). The accuracy (*R*^2^ = 0.99) of the calculated coverage ratio was validated against a corresponding benchmark dataset. The EasyPCC source code is released under GPL license with benchmark datasets of several different crop types for algorithm development and for evaluating ground coverage ratios.

## 1. Introduction

Given the growing demand for high-throughput phenotyping to support crop breeding, researchers have conducted experiments in fully-automated facilities, and they have been successful in assessing crop growth and performance using combinations of modern technologies, including genetic engineering, robotics, and machine learning [1,2,3]. In plant phenomics, image analysis is also considered a powerful tool for extracting phenotypic traits, both in controlled environments and in the field [4,5,6]. 

The plant canopy coverage ratio (PCCr) is a parameter often used to indicate plant growth status. It has been reported that the PCCr is strongly correlated with the leaf area index, canopy light interception, nitrogen content, and crop yield [7,8,9,10]. The PCCr can be estimated easily from images because it is defined as the percentage of the orthogonal projection area relative to the area of crop foliage in the horizontal plane. Several tools have been developed and proven efficient, in comparison with traditional methods, for estimating PCCr, e.g., “VitiCanopy”, “Canopeo”, and “Easy Leaf Area” [11,12,13]. Generally, in crop studies, digital images are taken using standard consumer or smartphone cameras. These images are then segmented into vegetation and background using thresholding techniques, following which the PCCr is estimated by counting the pixels in each group. However, existing tools are unsuitable for images acquired under different lighting conditions. This is because, even for the same scene, the color values of digital image pixels vary as the light conditions change, which means different user-defined thresholds could be required for each image. To address this problem, our previous study proposed an effective and efficient method (i.e., a decision-tree-based segmentation model, DTSM) [14] to extract vegetation regions from field-derived images taken under diverse natural lighting conditions. This method has been used successfully for wheat, soybean, tomato [15,16,17], and even gravels in the desert (Mu et al., unpublished). However, the computational run time is problematic when large numbers of high-resolution images are involved. 

Field-based platforms used for high-throughput phenotyping, e.g., moving vehicles, drones, or field-based scanning platforms, normally collect hundreds or thousands of images during a single day and the number increases steadily throughout the experimental period. It is a considerable challenge to handle and process each of these images individually. Therefore, an easily-implemented simple tool is required to estimate the PCCr for a high-throughput phenotyping platform. In this paper, we present a machine-learning-based tool called EasyPCC, which is based on the DTSM and designed to estimate the PCCr. EasyPCC is available as an R package, MATLAB^®^ (The Mathworks, Natick, MA, USA)-based executable program, and as source code in both languages for those users willing to make modifications for their own specific requirements. The tool contains functions including image sorting, user-defined DTSM generation, image processing, and PCCr derivation.

## 2. Implementation 

EasyPCC is characterized by the following features. (1) The functions are easily operated with the aid of a graphical user interface (GUI) (MATLAB^®^ version); (2) No special computer devices are required for image processing; (3) A large number of images (e.g., >1000) can be handled with just several clicks; (4) The PCCr can be stored in “*.csv” format files and it can be exported easily for analysis using other software; and (5) the source code is available for other users to manipulate.

### 2.1. The Basic Idea Behind EasyPCC

**Figure 1:**
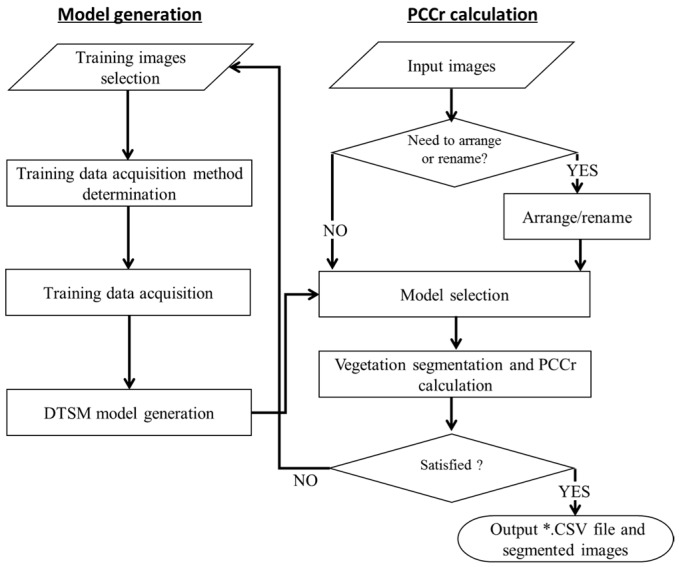
Workflow of EasyPCC.

EasyPCC comprises two important components: (1) model generation through the acquisition of a training dataset from the raw images, and (2) the segmentation of the vegetation from the background of the image, and the subsequent estimation of the PCCr. Figure 1 illustrates the workflow of EasyPCC. The details of each step are introduced in the following paragraphs.

### 2.2. Training Image Selection and Training Data Acquisition

EasyPCC is distributed with training images and data for wheat, paddy rice, sorghum, cotton, soybean, and sugarcane; however, users can also build their own datasets to train the DSTM. The core algorithm of EasyPCC is a DTSM and, therefore, the acquisition and input of “good” training data is the most important part of the entire workflow for providing the characteristics that are optimized for learning and generalization. A “good” training dataset for the DTSM is considered one that can train the model to segment only the vegetation from field-derived images taken under variable lighting conditions. We suggest that training images should cover heterogeneous natural lighting conditions. From the selected training images, the training data that include nine color features are collected and categorized into two classes: vegetation and background. The nine color features (R, G, B, H, S, V, L*, a*, and b*) are defined in three widely-used color spaces (RGB, HSV, and CIELab). EasyPCC provides two methods for collecting the training data, which are “line drawing” and “patch gathering”. 

#### 2.2.1. Line Drawing Method

**Figure 2:**
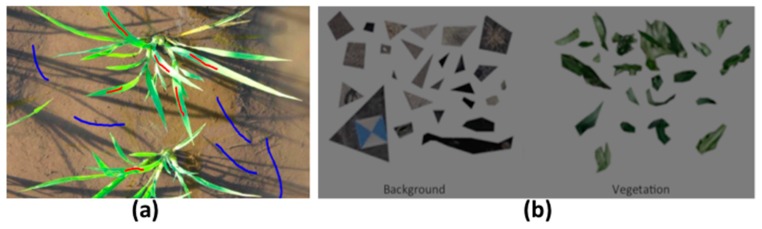
Two images for collecting training data: (**a**) a paddy rice image for the line drawing method; and (**b**) a sorghum image for the patch gathering method.

Users can open the selected training images and draw lines on the vegetation and background regions using left and right mouse clicks, respectively. The nine color features of the pixels on the lines are recorded automatically and saved as “*.csv” files with file names formatted as “vegetation + image Name + create time” and “background + image Name + create time”. It is highly recommended that all possible different parts of training images should be selected (e.g., the shadowed and spectrally-reflected parts of the same image, Figure 2a).

#### 2.2.2. Patch Gathering Method

This method allows users to create two blank training images as containers, to crop patches of vegetation and background targets into the containers, and then save them as “*.png” images with an alpha channel (Figure 2b). Finally, a function is provided that can read the two training images to extract the nine color features and build the training model.

### 2.3. Model Generation and PCCr Calculation

**Figure 3:**
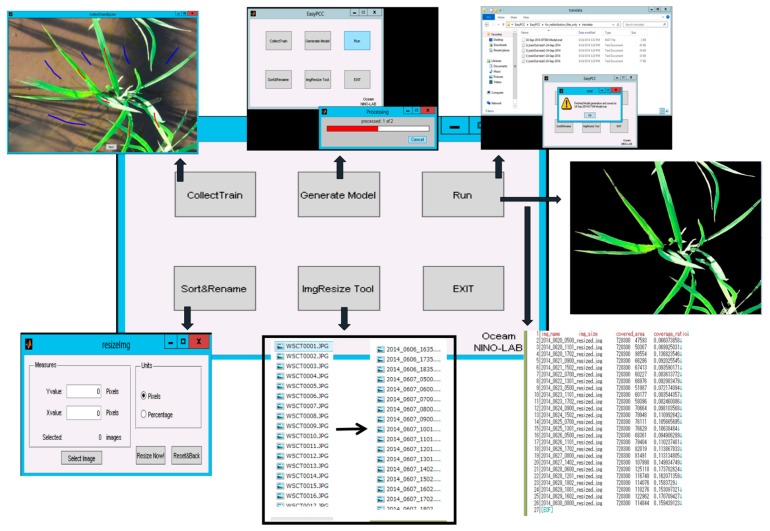
A demonstration of EasyPCC.

After the collection of training data, the other steps are simply followed via a few mouse clicks and/or function calls. Users can generate a segmentation model that fits their own data and then estimate the PCCr automatically. The PCCr is stored in “*.csv” format files which can be exported easily for analysis by other software. Figure 3 shows an example of how to implement EasyPCC under the MS Windows^®^ operating system. The detailed information and manuals of different versions of the EasyPCC package can be found in Supplementary Materials S1–S3.

## 3. Beta Testing of EasyPCC, the Experiment, and Results

**Figure 4:**
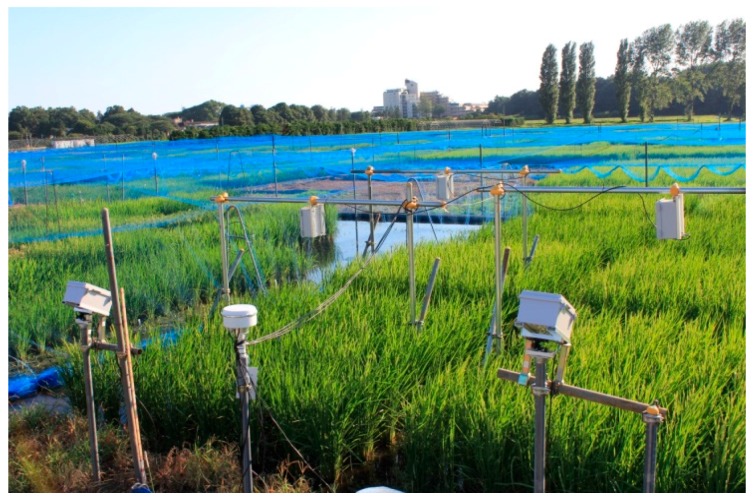
Field monitoring system.

Beta testing of EasyPCC was conducted using a Japonica rice variety *Kinmaze*. Seeds were sown on 26 April 2013 and seedlings were transplanted on 31 May 2013 at the Institute for Sustainable Agro-ecosystem Services, University of Tokyo, Japan (35°44′21.7″ N, 139°32′31.9″ E). The images were acquired using a field monitoring system, as shown in Figure 4, which involved a Canon EOS Kiss x5 digital camera with an EF-S18-55 mm lens mounted above the rice crop (2 m). Time-lapse images were taken at 1-h intervals and transmitted to a free webserver (http://www.flickr.com) via the 3G network [18]. Testing included evaluation of the software performance and analysis of the effects of the training data on the accuracy of the PCCr.

### 3.1. Experiment and Matierals

**Figure 5:**
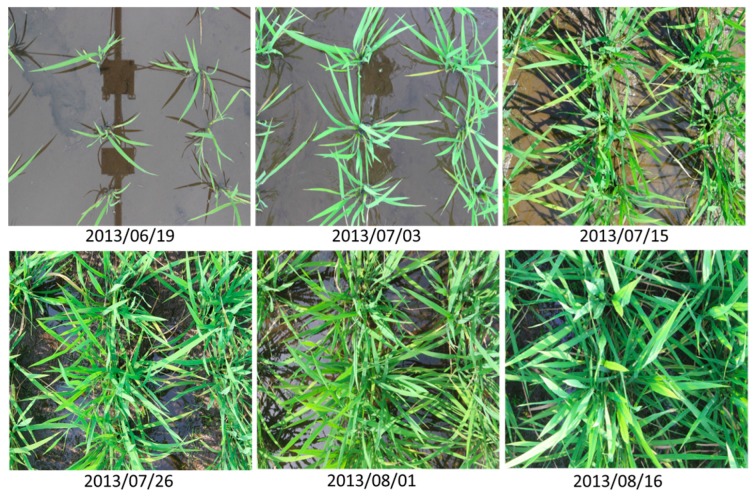
Examples of field images obtained throughout the period of observation (note the lighting conditions differ in each image).

Datasets of images for evaluation of the PCCr were acquired daily from 19 June (20 days after transplanting) to 16 August (approximately one week before heading) from about 08:00 to 16:00 local time. To prevent damage by foraging birds, a blue net was placed over the entire field on 30 July. Figure 5 shows six different images obtained at six growth stages. In the experiment, 10 training images were selected manually from the complete image sets taken during the initial and early–middle growth stages (i.e., between 20 and 78 days after transplanting), with consideration of the variations of weather and lighting conditions. Then, training data were carefully selected for two classes (vegetation and background) from each image using the line drawing method.

### 3.2. Effect of Training Data on PCCr Accuracy

**Figure 6:**
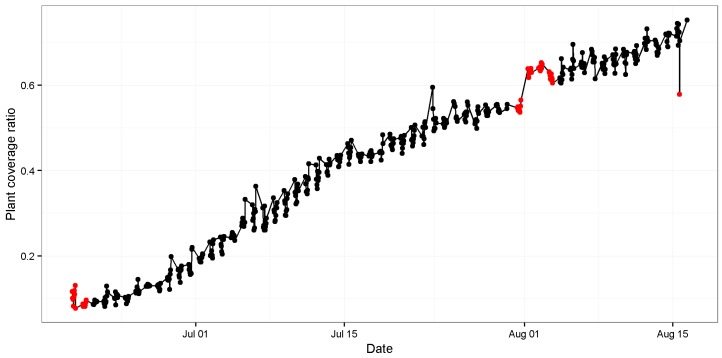
PCCr of rice paddies. The *x*-axis indicates the number of days after transplanting, and the *y*-axis shows the canopy coverage ratio. The black dots represent the calculated PCCr values based on EasyPCC, and the red dots represent PCCr values derived manually from the corresponding benchmarked images (considered the true values). See the text for further details.

**Figure 7:**
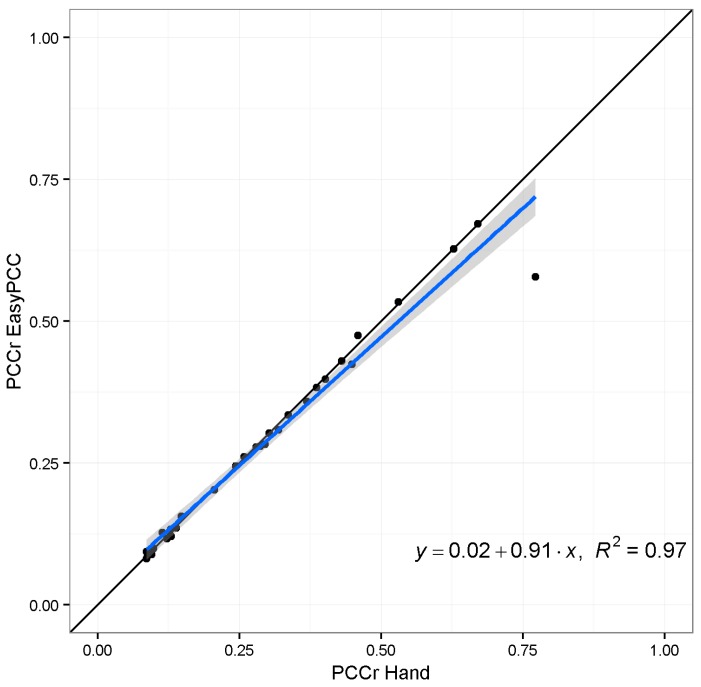
Relationship between the PCCr calculated by EasyPCC and the manually-derived true values. The solid black and blue lines represent a 1:1 relationship and the linear regression, respectively.

The estimates of PCCr were derived using EasyPCC, as shown in Figure 6. It is evident that the canopy coverage ratio following transplanting increases with time. High positive correlation was found between the DTSM-derived values and the true values of the canopy coverage ratios (*R*^2^ = 0.987, slope = 0.96; Figure 7). 

**Figure 8:**
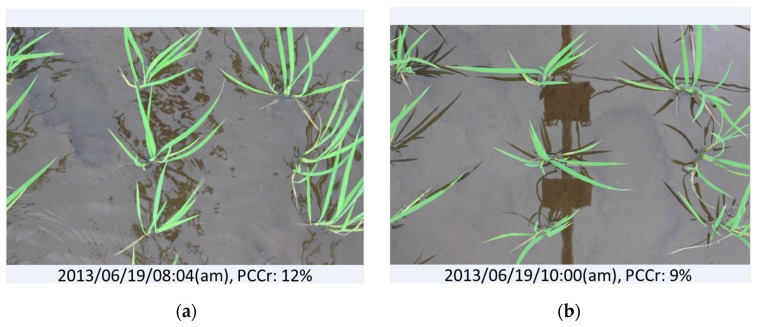
Wind strongly affects evaluation of the PCCr from images: (**a**) windy conditions and (**b**) non-windy conditions. The shape of the canopies varies dramatically.

**Figure 9:**
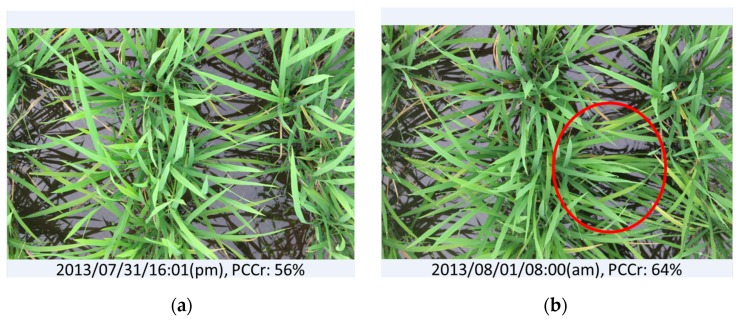
Physical disturbance of vegetation can affect the evaluation of PCCr from images: (**a**) normal vegetation and (**b**) vegetation flattened slightly by an unknown cause.

Three suspicious data points resulted from the evaluation of vegetation cover by EasyPCC (the red dots in Figure 6). The first dubious point suggested that the canopy coverage ratio on day 20 was greater than on day 21; a result attributable to strong wind, as shown in Figure 8. Another dubious point in the middle of the graph suggested that the coverage ratio on day 63 (image: “2013/07/31/16:01(p.m.)”) increased suddenly and then declined two days later at day 65. This temporary flattening of several stems was caused by an unknown source (possibly raccoon dogs) and it persisted for two days, as shown in Figure 9. The final dubious point suggested that the canopy coverage ratio decreased suddenly from 73% to 58%. The reason was that the raw images were taken 77 days after transplanting when the rice plants had grown and developed many overlapping leaves. Due to the direction of the sunlight, some of the leaves nearest the ground were covered by strong shadows. This weakened the color features in the digital images and, thus, reduced the ability of EasyPCC to isolate the vegetation accurately. 

**Figure 10:**
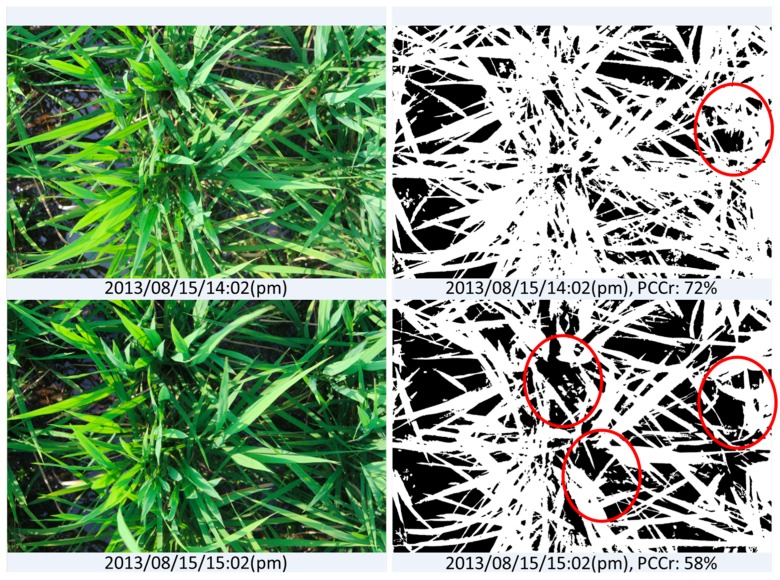
Dark shadows such as those in the lower-left photo can result in an underestimation of PCCr.

**Figure 11:**
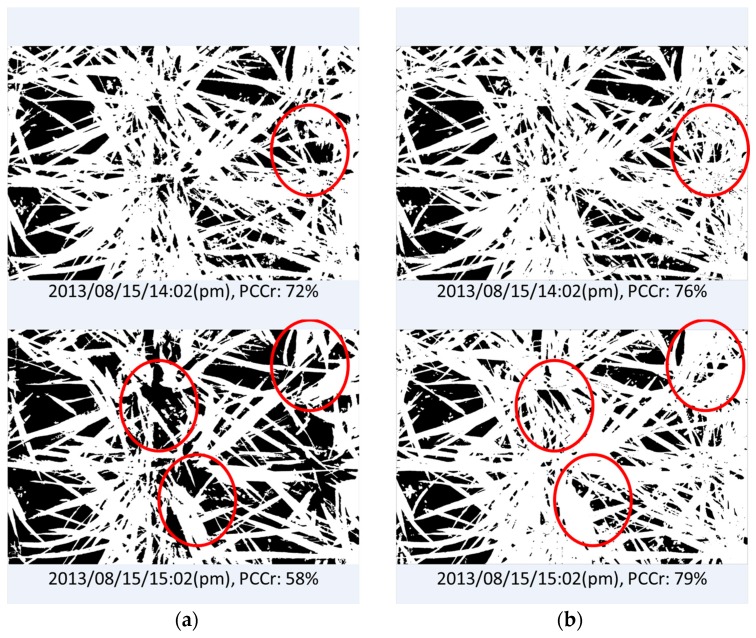
Comparison of the PCCr estimation by different EasyPCC models: (**a**) results obtained using the model trained without the training data from dark regions and (**b**) results obtained using the model trained with the training data that had dark regions added.

**Figure 12:**
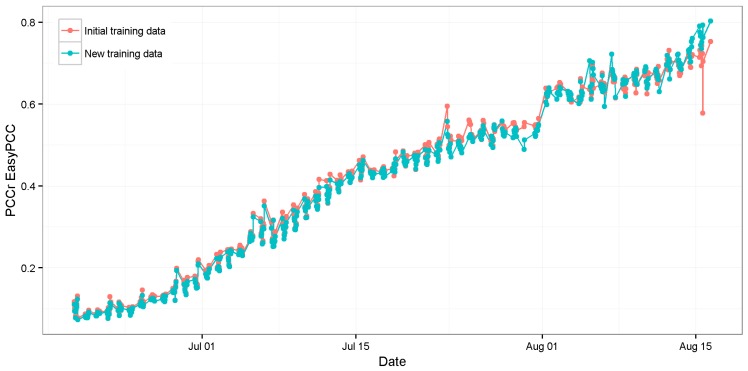
Comparison of rice crop canopy coverage ratios calculated by the EasyPCC model trained without (red dots) and with (blue dots) the newly-added training data from dark crop regions.

**Figure 13:**
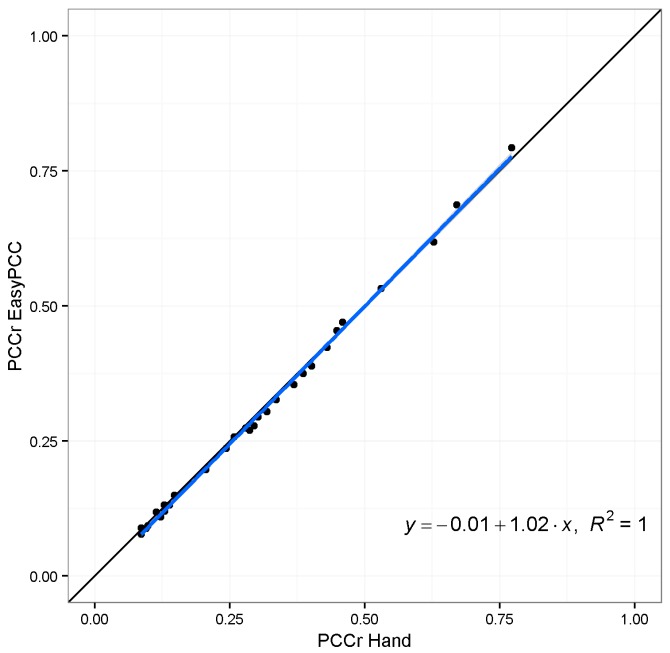
Relationship between the PCCr estimated by the EasyPCC model trained with the newly-added training data and the manually-derived true values. The solid black and blue lines represent a 1:1 relationship and the linear regression, respectively.

The evaluation results of image “2013/08/15/15:02(p.m.)” and another taken 1 h previously (“2013/08/15/14:02(p.m.)”) are shown in Figure 10. The shadows in both images caused significant underestimation of vegetation coverage; however, image “2013/08/15/15:02(p.m.)” lost more vegetation pixels because of the larger dark areas. This error is acceptable because we did not use any vegetation pixels from the shadowed parts of those images as initial training data, i.e., the model classified those dark pixels as belonging to the background class because the color values were close to the training data of background elements. This strong effect of the selection of the training data is the weakness of a machine-learning-based approach. The training data are selected manually, which makes it difficult to include all possible dubious cases to address the mentioned underestimation problem. To overcome the weakness, we added 12,000 pixels selected from the dark regions of the crop image to the training dataset for the vegetation class, and trained the model again with the new training data. Figure 11 and Figure 12 show the comparison of the segmentation and the PCCr using the newly-constructed model and the former model without the training data from the dark regions. The new model improved the segmentation result for image “2013/08/15/15:02(p.m.),” with the true coverage ratio of 76%, raising the estimated value from 58% to 79%, whereas the result for image “2013/08/15/14:02(p.m.)”, which does not have the dark shadowed region, achieves almost the same accuracy. Furthermore, the values obtained at the early growth stage also remained similar to before (Figure 12). The value of R^2^ between the DTSM-derived values and the true values was as high as 0.99 (Figure 13).

## 4. Conclusions

In this paper, we released a tool (EasyPCC) and source code (Supplementary Materials S1–S3) for high-throughput measurements of plant canopy coverage ratios under field conditions. The tool can be operated easily without the need for any special image processing techniques. The beta testing results demonstrated the high accuracy achievable by EasyPCC in evaluating the PCCr from digital images taken under natural field conditions. The results can be output as “*.csv” format files, which can be exported easily for analysis using other software packages, such as Excel^®^ or R. The robustness of this tool to the influences of various environmental factors, such as wind and animal destruction, was also demonstrated. We suggest running the software once for an entire image dataset to identify and discard poor images whose evaluated PCCrs are markedly different from the others. The time cost is about 30 s per 6 MB image when using an Intel i7 CPU and 16 GB memory computer, we suggest using parallel computing if the user has a multi-core CPU or cluster, allowing a number of images (depending on the numbers of cores/size of the cluster) to be processed at the same time. 

**Figure 14:**
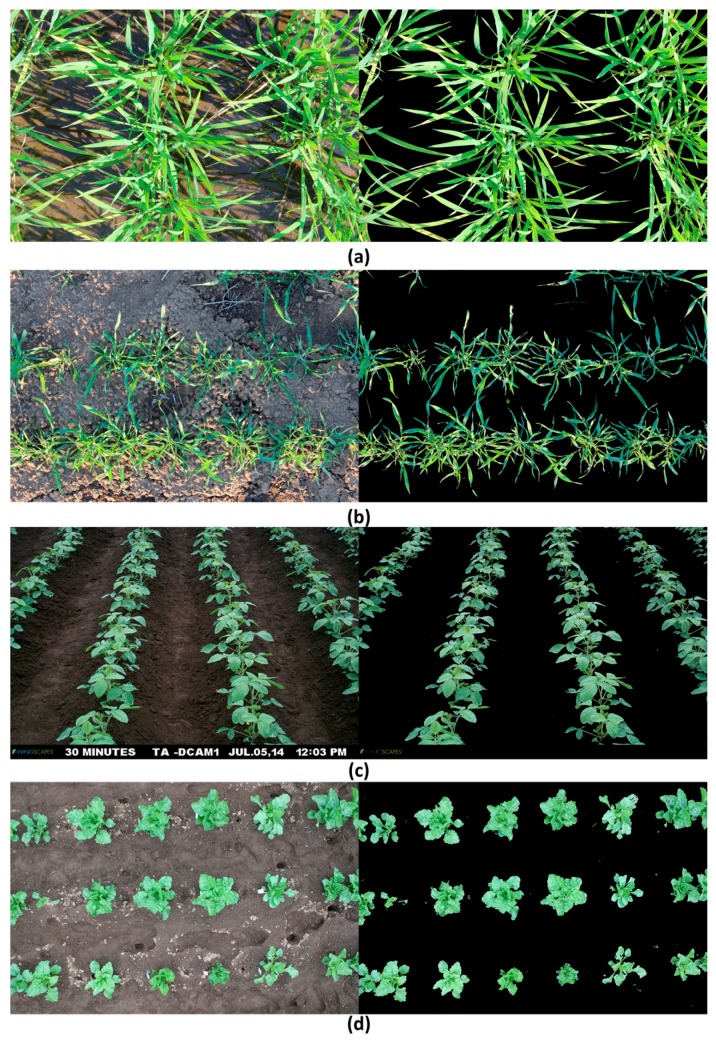
Demonstration of applying EasyPCC on images of different crops taken by ground camera or vehicles. (**a**) Paddy rice; (**b**) Wheat; (**c**) Soybean; (**d**) Sugar beet. The left side show the original images and the right side show the EasyPCC processed images which the background pixels were painted to black.

**Figure 15:**
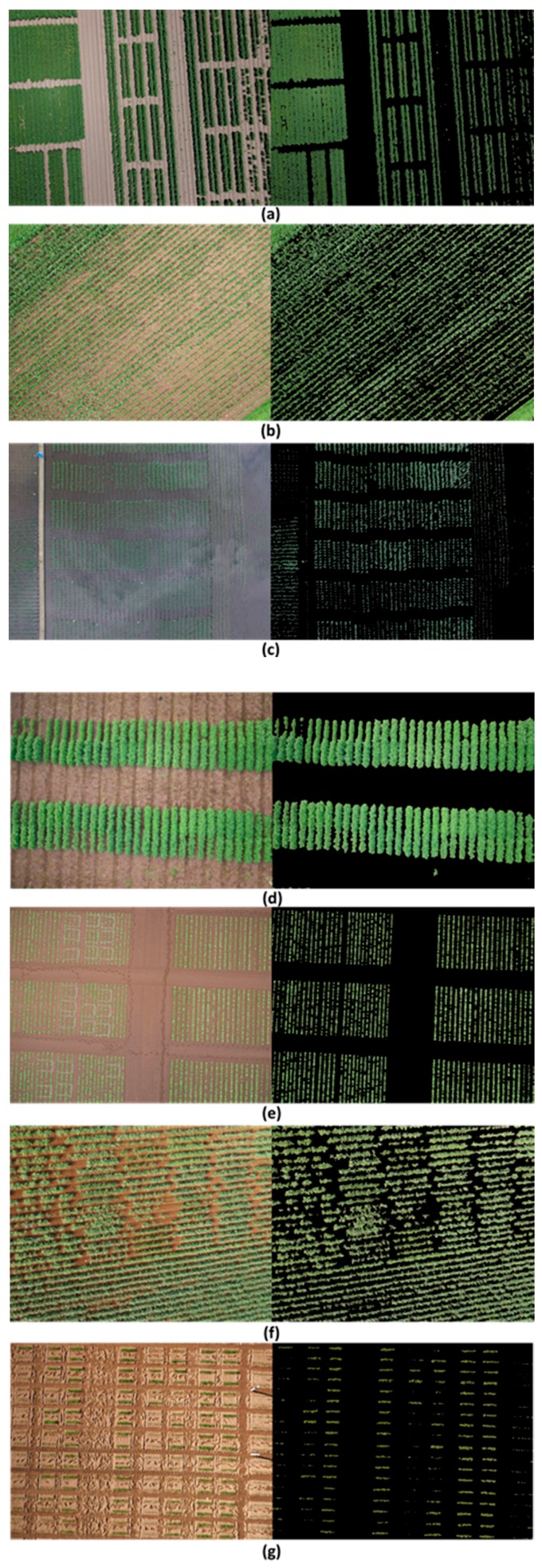
Demonstration of applying EasyPCC on images of different crops taken by UAV. (**a**) cotton; (**b**) Maize; (**c**) Paddy rice; (**d**) Soybean; (**e**) Sugar beet; (**f**) Sugarcane; (**g**) Wheat. The left side show the original images and the right side show the EasyPCC processed images which the background pixels were painted to black.

Currently, various types of field monitoring systems for field phenotyping exist, e.g., field servers, field cameras, moving vehicles, and drones, which are intended for screening large plant collections. Such systems collect large numbers of images throughout the growth cycle of the target crop, and EasyPCC has been proven the appropriate tool for handling such volumes of data with high efficiency and accuracy. Figure 14 and Figure 15 demonstrated some examples of applying EasyPCC on different crops taken by ground cameras/vehicles and UAVs.

The application of image analysis technologies to field-based plant phenotyping is still an emerging research topic, and we anticipate that additional algorithms will be developed in this field in the near future. However, evaluating the image segmentation accuracy of an algorithm is not easy, e.g., true values are needed. This means that for a given test image, a correct completely segmented image should be provided. This is normally achieved via hand labeling or drawing, processes that are both labor intensive and costly. Moreover, because the process is manual, different observers might produce different true values that add additional error to the evaluation of the segmentation algorithms. To avoid these issues, many databases already exist that provide true values of images, such as “The Berkeley Segmentation Dataset and Benchmark” [19], and the “Segmentation evaluation database” [20]. Researchers can download test images and check the performance of their own algorithm. We have collected a large number of field images taken by different field phenotyping platforms, some of which have been carefully labeled by hand. We would like to share those images as the start of a dataset, and we will continue to contribute further images to this dataset to aid other researchers who might wish to evaluate their own proposed algorithms (Supplementary Materials S5).

## Supplementary Materials

The following are available online at https://www.dropbox.com/s/wl6gql2w5779dyn/S1_Windows%C2%AEbased%20software%20EasyPCC.zip?dl=0; https://www.dropbox.com/s/t50d8ulo2rmawwi/supplementaryS2-S4.zip?dl=0 and https://www.dropbox.com/sh/v66jwmy8xfqm0xp/AAA9oIThaq7BGGLLZWzd-ez5a?dl=0. S1: Windows^®^-based software EasyPCC; S2: Matlab^®^-based source code of EasyPCC; S3: R package of EasyPCC; S4: howToUseEasyPccRversion.docx; S5: Dataset including field images and hand labeled images of crops.
